# Extensive pyomyositis secondary to paronychia-related MRSA infection: A case report

**DOI:** 10.1097/MD.0000000000028431

**Published:** 2022-01-14

**Authors:** Ying-Chi Wong, Hsi-Chih Chen, Chou-Cheng Lai

**Affiliations:** a Division of Pediatric Gastroenterology, Department of Pediatrics, Taipei Medical University Hospital, Taipei, Taiwan; b School of Medicine, Taipei Medical University, Taipei, Taiwan; c Division of Nephrology, Department of Medicine, Tri-Service General Hospital, Taipei, Taiwan; d School of Medicine, National Defense Medical Center, Taipei, Taiwan; e School of Medicine, National Yang-Ming University, Taipei, Taiwan; f Division of Infectious Diseases, Department of Pediatrics, Taipei Veterans General Hospital, Taipei, Taiwan.

**Keywords:** methicillin-resistant strains of *Staphylococcus aureus*, paronychia, pyomyositis

## Abstract

**Rationale:**

Pyomyositis is characterized by an insidious and multifactorial inflammatory process, which is often caused by hematogenous pathogen. Predisposing risk factors include immunodeficiency, diabetes, malignancy, or trauma. The spectrum of clinical presentation depends on disease severity, typically presented by fever and hip pain. We hereby present a case with extensive pyomyositis secondary to chronic paronychia infection.

**Patient concerns:**

A 14-year-old immunocompetent male presented with fever and hip pain. The patient was initially surveyed for common infectious etiologies prior to the presentation of acute limping, which led to image confirmation of extensive pyomyositis.

**Diagnosis:**

The patient presented with acute pain in the right hip accompanied by headache, myalgia of the right leg, and intermittent fever for a week. Physical examination disclosed limping gait, limited range of motion marked by restricted right hip flexion and right knee extension, and chronic paronychia with a nail correction brace of the left hallux. Diagnosis of pyomyositis was confirmed by magnetic resonance image. Methicillin-resistant strains of *Staphylococcus aureus* was isolated from the patient's blood and urine cultures within 2 days of collection. The same strain was also isolated from the pus culture collected via sonography-guided aspiration.

**Interventions:**

Antibiotics treatment with oxacillin, teicoplanin, daptomycin, and fosfomycin were administered. Sonography-guided aspiration and computed tomography-guided pigtail drainage were arranged, along with nail extraction of his left hallux paronychia prior to discharge. Oral antibiotics fusidic acid was prescribed. Total antibiotics course of treatment was 4 weeks.

**Outcomes:**

The patient gradually defervesced and was afebrile after drainage. Followed limb doppler sonography showed regression of the abscess at his right lower limb. Gait and range of motion gradually recovered without sequelae.

**Lessons:**

Ambulation and quality of life are greatly affected by the inflammatory process of pyomyositis. Detailed evaluation of predisposing factors should be done, even in immunocompetent individuals. Timely diagnosis is vital to successful treatment.

## 1 Introduction

Classically reported in tropical regions and often referred to as “pyomyositis tropicans”,^[1]^ pyomyositis is characterized by intramuscular abscess often caused by hematogenous pathogen.^[2]^ Aside from common predisposing risk factors such as underlying condition of diabetes, immunodeficiency, or malignancy, migration of related pathogen from a distant infectious site along with localized microtrauma of the affected skeletal muscle should also be considered.^[3]^ The spectrum of clinical presentation depends on disease severity, typically when a child presents with fever and hip pain, as compatible with commonly involved muscle groups of the pelvis and lower limbs.^[4]^ Imperative treatment goals include early diagnosis, a comprehensive survey for possible infection sources, prompt antibiotics administration, and timely abscess drainage.

We hereby present a case of extensive pyomyositis secondary to chronic paronychia infection, which is the first case reported in current literature.

## 2 Case presentation

**Figure 1:**
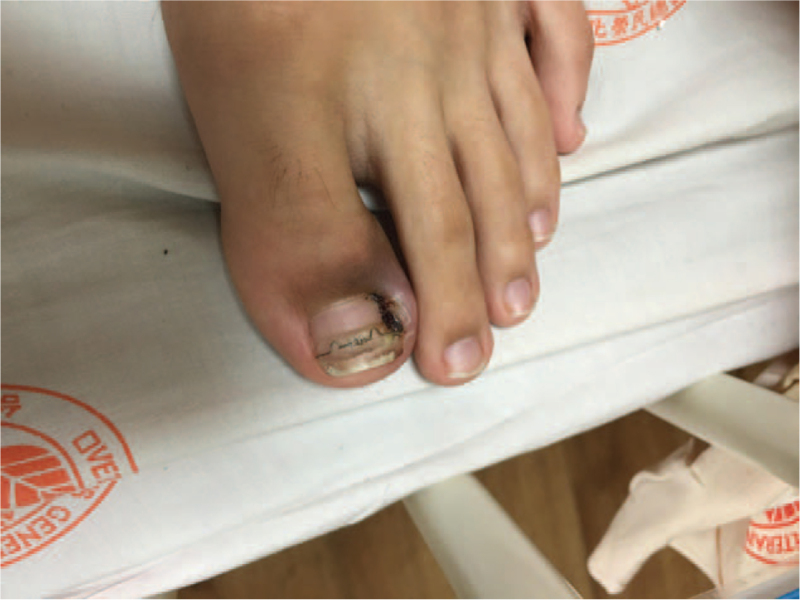
Chronic paronychia of the left hallux, treated with nail correction brace.

A 14-year-old boy presented to our pediatrics emergency department in January 2017, with a history of acute pain in the right hip accompanied by headache, myalgia of the right leg, and intermittent fever for a week. Consent for publication of patient information including images was obtained from the patient and the patient's caretaker, his mother. Besides chronic paronychia caused by basketball sports trauma for 3 months, he had been previously healthy. He had first been seen for his fever at an orthopaedics local medical clinic and the pediatrics outpatient department of another secondary care hospital. Screening for influenzae was negative. Pyuria was found, therefore urinary tract infection was presumed. Despite treatment with several courses of oral antibiotics cephalexin, fever persisted. He then came to our pediatrics emergency department. His vital signs were as follows: temperature 39.9 °C, pulse 92/min, respiratory rate 19/min, blood pressure 134/74 mm Hg. His gait was affected with a limp, as well as limited range of motion marked by restricted right hip flexion and right knee extension. Other physical examination revealed chronic paronychia with a nail correction brace of the left hallux (Fig. 1), which was done at a nonmedical pedicure centre.

**Figure 2:**
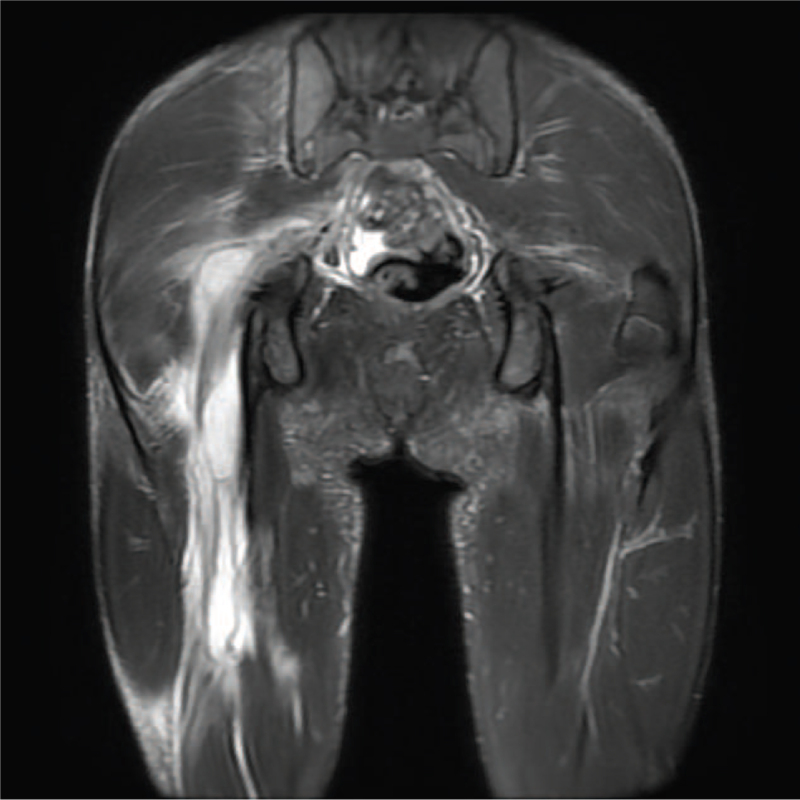
Magnetic resonance image (MRI) showing extensive abscess formation along right psoas muscle, right deep gluteal area, and right sciatic nerve.

The laboratory findings were as follows: white blood cell count 16,300/cumm, (normal range, 4500–11,000/cumm) with 88.4% neutrophils (normal range, 45%–75%), hemoglobin 11.1 g/dL (normal range, 14–18 g/dL), mean corpuscular volume 86 fL (normal range, 80–96fL), platelet count 444,000/cumm (normal range, 150,000–350,000/cumm), C-reactive protein 27.23 mg/dL (normal range, 0–0.5 mg/dL), erythrocyte sedimentation rate 75 mm/h (normal range, 0–10 mm/h). Pyuria was found by urinalysis (white blood cell/Pus 3–5/hpf; normal range < 5/hpf). Radiographic examination of the femur and hip disclosed no fracture and no dislocation. Magnetic resonance image study of the hip was ordered at the emergency department, which revealed extensive abscess formation along the right psoas muscle with extension to the right deep gluteal area and right sciatic nerve (Fig. 2). Methicillin-resistant strains of *Staphylococcus aureus* (MRSA) was isolated from the patient's blood and urine cultures within 2 days of collection. Antibiotics treatment with oxacillin was initiated, adjusted a day later to teicoplanin which was administered for 5 days.

Cardiac auscultation disclosed a grade III/VI systolic murmur at the left sternal border. Pediatric cardiologist was consulted. Echocardiography was unremarkable. On the second day of admission, swelling with palpation tenderness and localized heat progressed over his right popliteal region, with extension to his thigh and lower leg. To explore the retroperitoneal space, computed tomography scan disclosed abscess involvement compatible with magnetic resonance image findings, localizing the largest abscess at the right psoas muscle, over 3.2 cm × 4.1 cm × 8.9 cm in size, with extension to the right iliacus muscle, right piriformis muscle, right quadratus femoris and adductor muscles of the upper thigh. No retroperitoneal nor bony lesions were visible. Pediatric surgeon was consulted. Due to the extent of involved areas, operative debridement was not suitable. Sonography-guided aspiration was done on the second day of admission. MRSA was isolated from aspirate pus culture. Followed lab data disclosed reduced C-reactive protein. However, due to persistent fever, antibiotics were adjusted to daptomycin and fosfomycin on the seventh day of admission, administered for 15 days. Computed tomography-guided pigtail drainage was performed a week after admission. Drainage content was purulent, moderate in amount. He gradually defervesced and was afebrile 2 days after drainage. Followed limb doppler sonography examination showed residual abscess at the right gluteal region and regression of the abscess at his right lower limb. Nail extraction of his left hallux paronychia was arranged prior to discharge, on the third week of admission. Oral antibiotics fusidic acid was prescribed after discharge. Total antibiotics course of treatment was 4 weeks. The patient's gait and right lower limb motility gradually recovered without sequelae.

## 3 Discussion and conclusions

As with all cases of infectious diseases, assessment of predisposing conditions, including immune status, malignancy, contact history, and further susceptible factors such as wounds or trauma are essential.^[5]^ Commonly, secondary pyomyositis is characterized by directly affecting structural sources of infection, whereas primary pyomyositis is often related with unexplained *S aureus* septicemia.^[6]^ Thorough survey for secondary causes are vital due to increased mortality associated with secondary cases as compared to primary cases.^[7]^

Our case, an immunocompetent teen, presented with extensive pyomyositis, which is thought to be the direct result of septicemia due to underlying cellulitis caused by chronic paronychia. The infectious process arises from disrupted skin barrier, which leads to pathogen entrance, resulting in MRSA infection. For the past 10 years, 2 cases in literature present the possible association of localized infection resulting in distant tissue abscess. The first case published by Cosentino et al,^[8]^ a 60 year old male had underlying paronychia, which was thought to cause MRSA septicemia, resulting in septic spondilodiskitis. In the second case presented by Lee et al,^[9]^ localized finger cellulitis in a 78-year-old immunocompetent woman led to cervical spinal epidural abscess. Additionally, abscess formation is likely to be associated with the disseminated nature of the pathogen, as in psoas abscess caused by invasive group A *Streptococcus* in an immunocompetent 9-year-old boy,^[7]^ and reported cases of complicated tuberculous psoas abscess associated with tuberculous spondylitis.^[10]^

Pathogenesis of pyomyositis is insidious and multifactorial, including tendency of the renowned cutaneous pathogen, MRSA, to cause septicemia, as well as pathogen seeding to distant tissues. Possible microtrauma further explains defective barrier of affected musculature to microbial toxins.^[3]^ In our patient, nail correction brace application to a toe affected with chronic paronychia at a nonmedical facility most likely contributed to the introduction of bacteria into the bloodstream, which no associated cases were published in current literature. Even in immunocompetent individuals, the possibility of direct consequence of septicemia leading to abscess formation of a distant tissue emphasizes the importance of source identification. Comprehensive infection survey, timely antibiotics administration and surgical drainage are vital.

## Acknowledgments

We thank the patient and his family for granting permission to publish this article.

## Author contributions

YCW and HCC researched data and wrote the manuscript. YCW, HCC, and CCL contributed to the discussion.

**Investigation:** Ying-Chi Wong, Hsi-Chih Chen.

**Supervision:** Chou-Cheng Lai.

**Writing – original draft:** Ying-Chi Wong.

**Writing – review & editing:** Hsi-Chih Chen, Chou-Cheng Lai.
